# Prevalence of iron deficiency among university kendo practitioners in Japan: an observational cohort study

**DOI:** 10.1186/s12970-020-00393-2

**Published:** 2020-12-07

**Authors:** Takahiro Nabeyama, Yosuke Suzuki, Kana Yamamoto, Michiko Sakane, Yoichiro Sasaki, Haruka Shindo, Morihito Takita, Masahiro Kami

**Affiliations:** 1 Faculty of Health and Sport Sciences, University of Tsukuba, 1-1-1 Tennodai, Tsukuba, Ibaraki, 305-8574 Japan; 2 Medical Governance Research Institute, Minato-ku, Tokyo, Japan; 3 Department of Internal Medicine, Navitas Clinic, Tachikawa, Tokyo, Japan; 4 Department of Reproductive, Developmental and Aging Sciences, Graduate School of Medicine, University of Tokyo, Tokyo, Japan; 5 Sakane M Clinic, Tsukuba, Ibaraki, Japan; 6 Graduate School of Comprehensive Human Sciences, University of Tsukuba, Tsukuba, Ibaraki, Japan

**Keywords:** Anemia, Martial art, Ferritin, Nutrient intake

## Abstract

**Background:**

Iron deficiency is widely recognized as being the cause of anemia in athletes, although iron status in athletes of *Kendo*, a traditional Japanese martial art based on swordsmanship and practiced as an educational sport, has not been widely investigated.

**Methods:**

We performed a health assessment on anemia and serum ferritin levels, along with nutrient intake evaluation, for Kendo practitioners in a university in Japan.

**Results:**

A total of 56 Kendo practitioners (39 male and 17 female) aged between 18 and 23 years participated in the study. No individuals exhibited WHO-defined anemia (less than 13 or 12 g/dL of hemoglobin levels in male or female), while hypoferritinemia (less than 30 ng/mL) was found in seven (41%) females but not in males. Significantly higher body mass index was found in the female athletes with hypoferritinemia compared to females with normo-ferritinemia in sub-analysis (median [interquartile range]; 25.6 [24.2, 26.9] versus 22.6 [21.7, 24.1], respectively. *p* < 0.05). No significant differences in the intake of iron were registered between males and females (with and without hypoferritinemia) using data from a food-frequency questionnaire survey.

**Conclusion:**

No apparent anemia was found in adolescent Kendo practitioners, although this study confirmed the presence of hypoferritinemia in several female athletes. Careful follow-up, involving both clinical and nutritional assessment, will be necessary for them to prevent progression into anemia. A future study with larger cohorts in multiple sites is warranted to assess the prevalence of iron deficiency for validation and, if necessary, to devise a strategy for improving the iron status in Kendo athletes.

## Background

Anemia occurs when the number of red blood cells and, consequently, their oxygen-carrying capacity in a human body is insufficient to meet the body’s physiological needs. Athletes experience three kinds of anemia [1]. So-called ‘sports anemia’ is a false condition resulting from an expansion of blood plasma volume as an adaptation for aerobic fitness [2]. Exertional hemolysis and resultant fatigue is a clinically insignificant condition because it is mild, rarely depletes iron, and seldom causes anemia [3]. Iron deficiency, however, has long been recognized as a problem among athletes, and there have been many suggestions for its etiology [4]. It is a true anemia, a common cause of fatigue and lowered performance in female athletes, but rarely occurs in men. Athletes, especially those in endurance events, tend to have relatively low hemoglobin levels as compared with the general population. So-called ‘sports anemia’ is a false anemia, seen in athletes who are actually aerobically fit because the total volume of red blood cells (RBC) in their body is normal. Their hemoglobin level is diminished because aerobic exercise expands their baseline plasma volume, thereby reducing the concentration of RBC. Exertional fatigue is a clear indicator of mild anemia. Loss of iron through rupturing of red blood cells during exertion almost never develops apparent anemia, but blood loss occasionally occurs in runners, particularly in long-distance athletes, with hematuria being the most visible form of blood loss. Increased demand from myoglobin in skeletal muscle cells, mechanical hemolysis, iron loss via sweat, gastrointestinal tract, and the exercise-induced inflammatory response has also been proposed as causal factors for iron deficiency [5–7]. Menstrual blood loss is another major cause of iron deficiency in female athletes [8]. The prevalence of iron deficiency in athletes varies, with accounts of up to 35% in females and 11% in males having been reported [4]. In Japan’s general population, Kusumi et al. reported that 18% of women aged between 20 and 29 were anemic, which is a higher prevalence than that seen in the United States (7.1% estimated for 15–29 year-old women) [9, 10]. A previous report analyzing the annual prevalence of anemia in Universiade athletes in Japan revealed that the anemia prevalence decreased to 1.7% (from 13.3%) between 1977 and 2011, although their actual iron deficiency was not evaluated [11].

The Japan Association of Athletics Federations (JAAF) issued a warning on the overuse of iron injection for young athletes in response to media reports describing that some long-distance runners in Junior and High schools were being given iron injections without an appropriate assessment of their anemia status or iron levels [12]. Iron supplementation should only be provided in cases of anemia and iron deficiency. It is not usual for any young athletes anywhere to have their iron status regularly monitored, unless they are exceptional or elite athletes. Consequently, basic data on anemia and iron storage in young athletes has not been carefully assessed and linked to performance levels, although female athletes on college teams in the US are regularly screened [13].

The Japanese fencing sport of *Kendo*, which is both artistic and strenuous, is viewed as a major martial art in Japan. Over 1.8 million Kendo practitioners are registered by the All Japan Kendo Federation, larger than the number of Judo athletes in the country [14]. However, few scientific publications on anemia in *Kendo* practitioners are available, even though a mechanical hemolytic anemia, called ‘march hemoglobinuria’ has been identified, arising from unique footwork called ‘*suri-ashi*’ often performed in *Kendo* [15, 16]. No study has been undertaken previously on Kendo practitioners to investigate the prevalence of anemia or iron status in the athletes. Herein, we report an evaluation of anemia, along with the status of iron storage, and nutrient intake in university athletes practicing *Kendo* in Japan.

## Methods

### Participants

A health check-up program for *Kendo* practitioners who affiliated with their athletic club of the University of Tsukuba was carried out in February 2019. All examination was performed by a Board-Certified Sports Medicine physician of the Japan Sport Association (Sakane M Clinic, Tsukuba, Ibaraki, Japan). The Institutional Review Board of the Medical Governance Research Institute (Tokyo, Japan) approved this study (approval Number; MG2018–12). All study participants gave written informed consent before check-ups. Body fat percentage was measured with a BC-118D body composition analyzer (Tanita Corp, Tokyo, Japan). The peripheral venous blood was collected at rest and shipped to the contract laboratory (SRL, Inc., Tokyo, Japan). The complete blood count, levels of serum ferritin, serum iron, total iron-binding capacity (TIBC) and transferrin saturation were measured. Nutrient intake was evaluated using a food-frequency questionnaire based on food groups (FFQg) [17].

### Definitions

Anemia was defined as being a hemoglobin level of < 13 g/dL for men or less than 12 g/dL for non-pregnant women, in accordance with the World Health Organization (WHO) criteria [18]. Hypoferritinemia was evaluated with multiple thresholds of less than 15, 30 and 50 μg/L of serum ferritin level [19, 20].

### Statistical analysis

The participant and hematological characteristics were summarized using descriptive statistics. The correlation with serum ferritin levels was evaluated with Spearman correlation coefficients. The two-group comparison was determined with the Mann-Whitney U test or Fisher’s exact test. Statistical significance was considered when a *p* value was < 0.05. All statistical analyses were performed with IBM SPSS Statistics Version 25 (IBM Corp, Armonk, NY).

## Results

### Participant characteristics

A total of 56 Kendo practitioners, consisting of 39 males (70%) and 17 females (30%), undertook a health check-up. Participant characteristics and their hematological outcomes were summarized by gender (Table 1). The body fat percentage (BF%) in females was significantly higher than in males (median [interquartile range]; 26.6 [23.8–29.1] versus 16.3 [13.5–18.4] %, *p* < 0.001) while no significant difference was seen in body mass index (BMI). Proportions of both previous history and iron supplement use were higher in females than males, but the differences did not reach statistical significance. No females were pregnant.

**Table 1:** Participant characteristics

Variables	Total (*n* = 56)	Male (*n* = 39)	Female (*n* = 17)
*Participant Characteristics*			
Age (years)	20 [20, 22]	20 [20, 22]	20 [19, 21]
Years of sports/athletics	15 [13, 17]	15 [13, 17]	15 [13, 17]
Body mass index (kg/m^2^)	24.4 [23.3, 26.5]	24.7 [23.5, 26.6]	23.8 [22.3, 26.9]
Body fat percentage^‡^	18.3 [14.8, 23.9]	16.3 [13.5, 18.4]	26.6 [23.8, 29.1]
Previous history of anemia	4 (8)	1 (3)	3 (19)
Previous use of iron supplements	11 (21)	6 (16)	5 (31)
*Anemia status*			
Prevalence of anemia	0 (0)	0 (0)	0 (0)
Erythrocyte count (× 10^4^/μL) ^‡^	496 [469, 554]	505 [487, 541]	444 [425, 473]
Hemoglobin (g/dL) ^‡^	14.8 [14.0, 15.8]	15.1 [14.7, 16.1]	13.4 [12.7, 14.9]
Hematocrit (%)^‡^	45.7 [43.6, 48.0]	46.8 [44.8, 48.4]	41.7 [39.9, 44.5]
MCV (fL) ^†^	93 [90, 94]	92 [90, 94]	94 [92, 96]
MCH (pg)	30.1 [29.4, 30.6]	30.1 [29.4, 30.4]	30.2 [29.7, 30.8]
MCHC (g/dL) ^†^	32.6 [31.9, 33.1]	32.8 [32.4, 33.3]	31.9 [31.6, 32.5]
*Iron metabolism*			
Ferritin (ng/mL) ^‡^	91 [49, 130]	108 [80, 144]	43 [25, 58]
> 100 and ≤ 300 ng/mL	25 (45)	24 (62)	1 (6)
> 50 and ≤ 100 ng/mL	16 (29)	11 (28)	5 (29)
> 30 and ≤ 50 ng/mL	8 (14)	4 (10)	4 (24)
> 15 and ≤ 30 ng/mL	5 (9)	0 (0)	5 (29)
≤ 15	2 (4)	0 (0)	2 (12)
Serum iron (μg/dL)	90 [65, 142]	91 [62, 144]	83 [72, 113]
TIBC (μg/dL)	333 [319, 355]	330 [319, 349]	347 [326, 386]
Transferrin saturation (%)	26.8 [18.8, 40.1]	27.8 [18.3, 45.5]	24.6 [20.5, 33.3]

Data are shown as median [lower and upper interquartile range] or number (percentage). *p < 0.05, ^†^*p* < 0.01, and ^‡^*p* < 0.001 for the comparison between male and female practitioners. Abbreviations: MCV: mean corpuscular volume; MCH: mean corpuscular hemoglobin; MCHC: mean corpuscular hemoglobin concentration; TIBC: total iron-binding capacity

### Prevalence of anemia

No participants who fulfilled the WHO anemia criteria were observed in this study (Table 1). Significant smaller counts of erythrocytes, lower concentration of hemoglobin and lower hematocrit values were seen in females compared to males (*p* for all comparisons < 0.001). The females showed significantly higher mean corpuscular volume (MCV) and lower mean corpuscular hemoglobin concentration (MCHC) than males (*p* < 0.01).

### Status of iron deficiency

Significantly lower levels of ferritin were observed in females compared to males (43 [25–58] versus 108 [80–144], *p* < 0.001). In contrast, serum iron concentration, TIBC, and transferrin saturation did not demonstrate significant differences between the gender groups. Two females (12%) and none of the males exhibited severe hypoferritinemia (< 15 ng/mL). The moderate hypoferritinemia between > 15 and ≤ 30 ng/mL was also seen in females only [*n* = 5 (29% of female practitioners)). No study participants who > 300 ng/mL of serum ferritin were observed. Two females were taking oral contraceptive pills (OCP), and their ferritin levels were 15 and 58 ng/mL.

Of note, inverse correlations of serum ferritin levels were seen with BMI and BF% in female practitioners (Spearman correlation coefficients *ρ* = − 0.46 and − 0.62. *p* = 0.06 and < 0.01, respectively) while males showed the positive correlations (*ρ* = 0.41 and 0.51. *p* < 0.01 for both correlations, respectively).

### Comparison of characteristics between hypo- and normo-ferritinemia in female practitioners

**Fig. 1:**
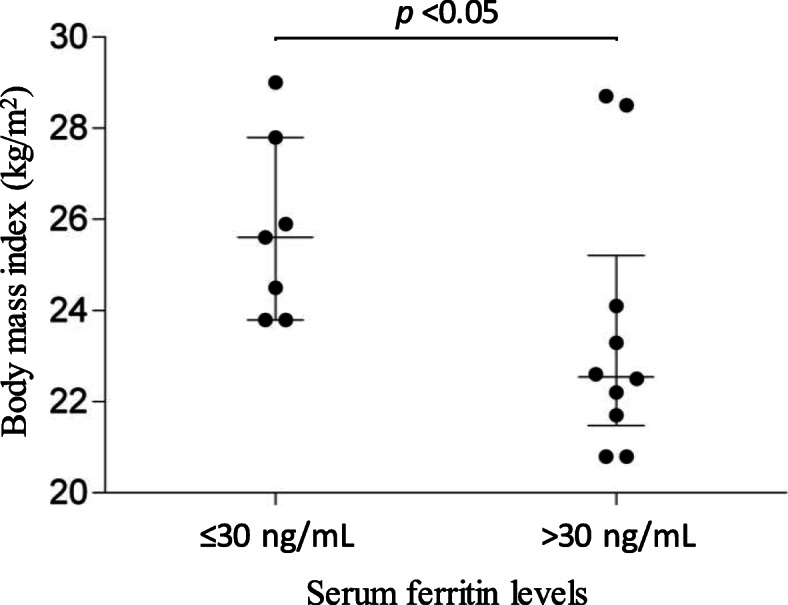
Distribution of body mass index classified by serum ferritin levels in female Kendo practitioners. Distribution of Body Mass Index (BMI) of female Kendo practitioners is shown with a dot plot classified by their serum ferritin levels. The median and the interquartile range are indicated. Significantly higher BMI was found in females with hypoferritinemia ≤30 ng/mL (*p* < 0.05)

We performed a sub-analysis to figure out the characteristics of female practitioners with hypoferritinemia (serum ferritin levels ≤30 ng/mL) when compared to those with > 30 ng/mL of serum ferritin (Table 2). Significantly higher BMI and BF% were found in hypo-ferritinemia individuals when compared to the normal group (*p* < 0.05 for comparison of BMI and BF%) (Fig. 1).

**Table 2:** Comparison of participant characteristics between hypo- and normo-ferritinemia in female practitioners

Variables	Hypo-ferritinemia (*n* = 7)	Normo-ferritinemia (*n* = 10)
*Participant Characteristics*		
Age (years)	20 [19, 21]	21 [20, 21]
Years of sports/athletics	12 [8, 15]	15 [14, 16]
Body mass index (kg/m^2^)*	25.6 [24.2, 26.9]	22.6 [21.7, 24.1]
Body fat percentage*	28.0 [27.4, 29.1]	24.1 [21.0, 24.8]
Previous history of anemia	2 (29)	1 (11)
Previous use of iron supplements	3 (43)	2 (22)
*Anemia Status*		
Hemoglobin (g/dL)	13.3 [12.8, 13.8]	13.6 [13.2, 14.2]
Hematocrit (%)	41.7 [39.9, 43.2]	42.2 [40.7, 44.6]
MCV (fL)	94 [93, 96]	94 [92, 95]
MCH (pg)	29.8 [29.4, 30.5]	30.2 [29.7, 30.8]
MCHC (g/dL)	31.9 [31.6, 32.3]	32.0 [31.7, 32.5]

Data are shown as median [lower and upper interquartile range] or number (percentage). *p < 0.05 for the comparison between hypo- and normo-ferritinemia groups

### Nutrient intake

The food intake of participants was evaluated with the FFQg, after classifying individuals into three groups of males and females with and without hypoferritinemia (Table 3). No significant differences in the intake of iron were seen among the three groups. The intake of iron, however, was high in the order of males, females without hypoferritinemia, and those with hypoferritinemia (7.4 [5.4–8.6], 6.7 [4.9–9.4], and 4.4 [3.4–7.4] ng/mL, respectively). Total energy intake and the intakes of water, carbohydrate, and manganese were significantly higher in males compared to females with and without hypoferritinemia (*p* < 0.05). Males also showed higher intakes of protein, sodium, magnesium, zinc, copper, molybdenum, vitamin B1, B6, niacin, and total fiber when compared to females with hypoferritinemia. No significant differences, however, were found between males and females without hypoferritinemia.

**Table 3:** Results of Food-Frequency Questionnaire Based on Food Groups (FFQg)

Variables	Male (*n* = 36)	Female-Normoferritinemia (*n* = 10)	Female-Hypoferritinemia (*n* = 7)56	*p* value		
				Male versus Female Normo-ferritinemia	Male versus Female Hypo-ferritinemia	Female-Normo- versus Hypo-ferritinemia
Energy (Kcal)	2343.5 [1873–2892]	1784.5 [1366–2062.75]	1269 [1015–2041]	< 0.05	< 0.01	*n.s.*
Water (g/d)	1222.4 [827.45–1308.25]	749 [552.15–1058.375]	642.7 [418.3–816.5]	< 0.05	< 0.01	*n.s.*
Protein (g/d)	74.5 [55.55–83.6]	62.85 [43.9–69.4]	37.8 [34.4–68.6]	*n.s.*	< 0.05	*n.s.*
Fat (g/d)	62.35 [46.125–77.125]	67.2 [50.25–71.675]	53.6 [36.1–79]	*n.s.*	*n.s.*	*n.s.*
Carbohydrate (g/d)	360.2 [251.275–435.825]	221.95 [188.775–273.125]	156 [110.8–277.7]	< 0.01	< 0.01	*n.s.*
Ash (g/d)	15.2 [12.675–17.275]	12.85 [10.475–17.6]	10.1 [7.7–14.8]	*n.s.*	*n.s.*	*n.s.*
Na (mg/d)	3601 [2651–4333.25]	2753.5 [2451–3606]	2478 [1931–3128]	*n.s.*	< 0.05	*n.s.*
K (mg/d)	1981 [1594.75–2375.75]	1744.5 [1316.75–2641.5]	1291 [872–2024]	*n.s.*	*n.s.*	*n.s.*
Ca (mg/d)	485.5 [400.25–583.75]	500 [338.25–575.5]	333 [250–523]	*n.s.*	*n.s.*	*n.s.*
Mg (mg/d)	247 [185.5–280.75]	196 [162.5–305.75]	135 [106–229]	*n.s.*	< 0.05	*n.s.*
P (mg/d)	1042 [759.5–1161.25]	967 [603–1126.25]	578 [488–1015]	*n.s.*	*n.s.*	*n.s.*
Fe (mg/d)	7.35 [5.375–8.6]	6.7 [4.85–9.35]	4.4 [3.4–7.4]	*n.s.*	*n.s.*	*n.s.*
Zn (mg/d)	8.95 [7.3–11]	7.35 [5.55–9]	4.2 [4–8]	*n.s.*	< 0.01	*n.s.*
Cu (mg/d)	1.25 [0.995–1.4075]	0.885 [0.6925–1.3825]	0.52 [0.45–1.04]	*n.s.*	< 0.01	*n.s.*
Mn (mg/d)	3.115 [2.39–3.8]	1.995 [1.6175–3.2]	1.13 [0.8–2.27]	< 0.05	< 0.01	*n.s.*
Iodine (μg/d)	394 [243.5–724.5]	432 [197.25–988.5]	249 [101–618]	*n.s.*	*n.s.*	*n.s.*
Se (μg/d)	69 [49.25–78]	58.5 [40–68.5]	40 [31–67]	*n.s.*	*n.s.*	*n.s.*
Cr (μg/d)	6 [4–7]	6.5 [3.75–8.25]	5 [3–7]	*n.s.*	*n.s.*	*n.s.*
Mo (μg/d)	208.5 [145.75–286]	137.5 [111.5–195]	68 [39–148]	*n.s.*	< 0.01	*n.s.*
Retinol (μg/d)	162 [110.25–240.75]	167.5 [94.5–268.25]	164 [106–228]	*n.s.*	*n.s.*	*n.s.*
β-Carotene (μg/d)	1820 [1189.25–2660]	2319 [873–3028.5]	2074 [1068–2357]	*n.s.*	*n.s.*	*n.s.*
β-Carotene equivalent (μg/d)	2080.25 [1350.475–3059.675]	2942.6 [1034.025–3397.6]	2343.1 [1307.2–2763.7]	*n.s.*	*n.s.*	*n.s.*
Vitamin D (μg/d)	3.6 [2.15–5.475]	3.4 [2.1–5]	1.9 [1.7–4.6]	*n.s.*	*n.s.*	*n.s.*
αTocopherol (mg/d)	6.65 [4.7–8.2]	6 [4.725–7.725]	4.8 [2.7–7.1]	*n.s.*	*n.s.*	*n.s.*
Vitamin K (μg/d)	150.6 [111.025–180.7]	157.95 [106.25–265.65]	159.5 [61.2–186.6]	*n.s.*	*n.s.*	*n.s.*
Vitamin B1 (mg/d)	1.2 [0.825–1.4]	0.9 [0.675–1.075]	0.6 [0.5–1]	*n.s.*	< 0.05	*n.s.*
Vitamin B2 (mg/d)	1 [1–1]	1 [1–1]	1 [1–1]	*n.s.*	*n.s.*	*n.s.*
Niacin (mg/d)	14.615 [10.5125–17.1225]	10.9 [9.11–13.78]	8.26 [5.86–12.3]	*n.s.*	< 0.05	*n.s.*
Niacin equivalent (mg/d)	27.755 [20.72–33.2175]	23.145 [17.2375–27.385]	15.5 [12.58–24.84]	*n.s.*	< 0.05	*n.s.*
Vitamin B6 (mg/d)	1.1 [0.8–1.2]	0.9 [0.675–1.175]	0.7 [0.4–1]	*n.s.*	< 0.05	*n.s.*
Vitamin B12 (μg/d)	3.415 [2.4175–5.13]	3.64 [2.365–4.035]	2 [1.21–4.72]	*n.s.*	*n.s.*	*n.s.*
Folic acid (μg/d)	204.45 [150.45–263.65]	220 [138.5–336.65]	174.6 [97.6–237.4]	*n.s.*	*n.s.*	*n.s.*
Pantothenic acid (mg/d)	6.3 [4.8–6.945]	5.565 [3.56–6.5275]	3.46 [2.79–5.78]	*n.s.*	*n.s.*	*n.s.*
Biotin (μg/d)	26.745 [23.9525–32.4075]	31.125 [19.03–41.8825]	23.02 [18.48–35.83]	*n.s.*	*n.s.*	*n.s.*
Vitamin C (mg/d)	46 [35–71.25]	52.5 [30.5–87.75]	40 [23–56]	*n.s.*	*n.s.*	*n.s.*
Saturated fatty acids (g/d)	18.215 [14.3775–25.56]	20.865 [18.44–24.8]	19.8 [11.03–24.9]	*n.s.*	*n.s.*	*n.s.*
Monounsaturated fatty acids (g/d)	23.3 [16.525–27.425]	23.365 [17.835–24.6425]	18.29 [13.25–27.98]	*n.s.*	*n.s.*	*n.s.*
Polyunsaturated fatty acids (g/d)	12.41 [10.1175–14.6375]	12.255 [8.6125–15.13]	9.98 [6.28–14.49]	*n.s.*	*n.s.*	*n.s.*
Cholesterol (μg/d)	315.5 [240.5–388.75]	372 [239.25–456.5]	291 [288–482]	*n.s.*	*n.s.*	*n.s.*
Soluble fiber (g/d)	2.7 [1.9–3.375]	2.45 [1.8–3.7]	1.8 [1.1–2.7]	*n.s.*	*n.s.*	*n.s.*
Insoluble fiber (g/d)	9.3 [6.325–10.7]	7.1 [5.375–10.85]	5.7 [3–8.1]	*n.s.*	*n.s.*	*n.s.*
Total fiber (g/d)	12.75 [9.35–15.4]	9.9 [7.825–15.55]	7.5 [4.3–11.5]	*n.s.*	< 0.05	*n.s.*
NaCl (g/d)	9.1 [6.75–11.025]	6.95 [6.25–9.125]	6.3 [4.9–7.9]	*n.s.*	*n.s.*	*n.s.*
Ethanol (g/d)	3.07 [0.23–11.93]	2.39 [0.11–4.01]	3.07 [0.46–7.39]	*n.s.*	*n.s.*	*n.s.*
Total Fat (g/d)	54.62 [40.3825–67.275]	58.88 [45.795–62.215]	48.1 [32–68.51]	*n.s.*	*n.s.*	*n.s.*
n-3 fatty acids (g/d)	1.74 [1.4025–2.4925]	1.725 [1.26–2.1125]	1.22 [0.87–2.04]	*n.s.*	*n.s.*	*n.s.*
n-6 fatty acids (g/d)	10.8 [8.55–12.175]	10.5 [7.425–12.9]	8.7 [5.4–12.4]	*n.s.*	*n.s.*	*n.s.*

Three male participants missed the FFQg. Data are shown as median [lower and upper interquartile range]. *P* values with the Kruskal Wallis test are shown

## Discussion

To our knowledge, this article is the first report of the presence of hypoferritinemia in *Kendo* practitioners in Japan. No Kendo athletes were anemic, according to the WHO criteria, while hypoferritinemia (< 30 ng/mL) was seen in 41% of the female participants. They should be carefully monitored to prevent the development of overt anemia. The present study is an observational study with a small cohort serving as a pilot survey, where the statistical assessment has limited power. Consequently, further research with a larger cohort is warranted to validate our findings.

Although we found no anemia in the Kendo practitioners studied, a previous report on elite young athletes found a low incidence (~ 7%) of anemia in both males and females [21]. The iron status, however, exhibited a significant difference between genders -i.e., a significant higher proportion of iron deficiency was observed in female athletes in our study, which is consistent with previous reports [21, 22]. The causes of gender differences in iron deficiency include menstrual blood loss and, consequently, inadequate iron intake in females [23, 24]. Less iron intake was seen in female practitioners, either with normoferritinemia or with hypoferritinemia, when compared to the males in this study although higher iron intake has been recommended for female athletes [25]. Another cause of the gender difference could be the biological response to gonadal sex hormone, erythropoietin and hepcidin - i.e., testosterone in males is beneficial for erythropoiesis [26, 27]. In contrast, estradiol in females was reported to induce transcription of hepcidin mRNA, which may associate with lower iron storage [28, 29]. Higher iron storage was shown in females who used the OCPs [30]. We were not able to evaluate the effect of OCP in iron status since only two female participants in our study took oral contraceptive pills.

Interestingly, the direction of correlation between ferritin levels and BMI or BF% in female athletes was the opposite of that in males. Males with higher BMI showed higher ferritin levels, consistent with previous studies [31, 32], and obesity-related inflammatory processes may be an explanation for such a relationship [33]. Conversely, an inverse correlation between ferritin levels and BMI has been reported in female adolescents [34]. Lower levels of serum ferritin in females with higher BMI in this study may suggest an insufficient or unbalanced dietary habit to supplement the higher demand [35].

Limitations in the present study include limited statistical power due to small cohorts, unknown bias by single-site research and lack of longitudinal observation since this study was performed as a pilot study. Future studies will include larger cohorts with multiple study sites to overcome such limitations. Biological evaluation with serum hepcidin concentration would help to explore the biological mechanism of iron deficiency in athletes. An educational lecture on iron deficiency was performed together with individual feedback on laboratory results after the study. A follow-up evaluation of this cohort will help determine the effect of such education on improving iron status.

## Conclusion

In conclusion, no university *Kendo* practitioners in this study showed anemia, according to the WHO definition of the condition, although hypoferritinemia of < 30 ng/mL was observed in 41% of females. This study is the first to report the prevalence of iron deficiency in adolescent Kendo practitioners and suggests the importance of careful evaluation of their iron status. Future research, with larger cohorts in multiple sites, will assess the prevalence of iron deficiency for validation, explore iron metabolism (including hepcidin), and help to devise a strategy improving iron status in the Kendo athletes.

## Data Availability

The datasets generated and analyzed during the current study are not publicly available due to the containment of participant identifiable information but are available from the corresponding author on reasonable request.

## Abbreviations

BF%: Body fat percentage
BMI: Body Mass Index
FFQg: Food-frequency questionnaire based on food groups
JAAF: Japan Association of Athletic Federations
MCHC: Mean corpuscular hemoglobin concentration
MCV: Mean corpuscular volume
mRNA: Messenger ribonucleic acid
OCP: Oral contraceptive pill
RBC: Red blood Cells
TIBC: Total iron-binding capacity
US: United States
WHO: World Health Organization
